# Association between apathy and regional cerebral blood flow in patients with idiopathic normal pressure hydrocephalus

**DOI:** 10.1186/2045-8118-12-S1-O4

**Published:** 2015-09-18

**Authors:** Hideki Kanemoto, Hiroaki Kazui, Takashi Suehiro, Shingo Azuma, Syunsuke Sato, Yukiko Suzuki, Kenji Yoshiyama

**Affiliations:** 1 Department of Psychiatry Osaka University Graduate School of Medicine, Japan

## Introduction

Apathy is a common symptom in idiopathic normal pressure hydrocephalus (iNPH). However, the neuroanatomical bases of apathy in iNPH has not been well examined. We assessed the relationship between improvement of apathy and change of regional cerebral blood flow (rCBF) after shunt surgery.

## Methods

We recruited 20 iNPH patients who had apathy before shunt surgery. They were tested about apathy with Neuropsychiatric Inventory (NPI) and cognitive function with Mini-mental State Examination (MMSE) and Frontal Assessment Battery (FAB). They were also evaluated quantitative rCBFs of 32 regions-of-interests (ROIs) with 123I-IMP single photon emission computed tomography (SPECT) using the autoradiography method. All the evaluations were conducted both before and 3 months after shunt surgery. They were classified into two subgroups; one was improved apathy (APA+) and another was not (APA-). The changes of rCBFs after the shunt were evaluated in each subgroup, respectively. In addition, we assessed the correlations between the changes of apathy and cognitive functions after shunt surgery.

## Results

Ten patients categorized in APA+ group. In APA+ group, rCBFs in 3 ROIs, bilateral anterior cingulate cortices (ACC) and right caudate nucleus, were significantly improved after shunt surgery. In APA-group, rCBFs in 2 ROIs, splenium of corpus callosum and right amygdala, were significantly improved after shunt surgery. A significant correlation was found between the changes of apathy score in NPI and score of FAB.

## Conclusions

Dysfunction of bilateral ACC and right caudate nucleus could cause apathy, and severity of apathy could be correlated with frontal dysfunction in iNPH. Caudate is known as a key structure of apathy related to disruption of cognitive processing. The results in the present study might indicate that iNPH led to apathy as a result of frontal dysfunction due to caudate impairment.

